# Adherence to guidelines for antibiotics used in the initial treatment of febrile neutropenia in patients with cancer: a study using health insurance claims database in Japan

**DOI:** 10.1186/s40780-025-00455-0

**Published:** 2025-06-06

**Authors:** Kanako Mizuno, Ryo Inose, Ryota Goto, Yuichi Muraki

**Affiliations:** Laboratory of Clinical Pharmacoepidemiology, Kyoto Pharmaceutical University, 5 Misasagi Nakauchi-Cho, Yamashina-Ku, Kyoto, 607-8414 Japan

**Keywords:** Febrile neutropenia, Medical claims database, Antibiotic

## Abstract

**Background:**

*Pseudomonas aeruginosa*, a causative microorganism of febrile neutropenia (FN), accounts for approximately 15% of bloodstream infections and is associated with a high mortality rate. Therefore, antibiotics with anti-*P. aeruginosa* activity should be administered appropriately during the initial treatment of FN. While other countries have examined guideline adherence and its associated factors in FN initial treatment, limited data are available on these aspects in Japan. This study aimed to evaluate adherence to FN treatment guidelines regarding antibiotics used in patients with cancer and identify factors associated with adherence using a Japanese health insurance claims database.

**Methods:**

This study used the JMDC hospital-based administrative claims database between April 2014 and August 2022 obtained from JMDC Inc. Hospitalized patients with cancer with a definitive diagnosis of FN were included in the study. FN cases were defined as patients who underwent bacteriological culture and identification test for blood in the same month as their first definitive FN diagnosis. The date of the first bacteriological culture and identification test for blood was considered the date of the first FN definitive diagnosis.

**Results:**

Among 31,947 patients diagnosed with FN, 12,008 underwent bacteriological culture and identification test for blood in the same month as their FN diagnosis. After applying exclusion criteria, 11,292 patients were included in the analysis. The overall adherence rate to FN treatment guidelines for initial antibiotic selection in Japan was 78.8% and remained stable over time, consistently above 75%. Factors significantly associated with guidelines adherence included patients with hematologic malignancies (OR: 1.117, 95% CI: 1.007–1.239). The study also identified trends in antibiotic use in initial treatment. The use of penicillin with beta-lactamase inhibitor significantly increased over time (*r* = 0.01621, *p* < 0.001), while carbapenem use significantly decreased (*r* = -0.00813, *p* < 0.001).

**Conclusion:**

The study revealed an FN guideline adherence rate of 78.8% in Japan, along with changes in antibiotic prescribing patterns, including a trend toward carbapenem-sparing strategies between 2014 and 2022. Continuous surveillance is necessary, as adherence rates and antibiotic selection may be influenced by future guideline revisions and antimicrobial stewardship initiatives.

**Supplementary Information:**

The online version contains supplementary material available at 10.1186/s40780-025-00455-0.

## Background

Febrile neutropenia (FN) is an adverse event in patients with cancer receiving chemotherapy and requires careful monitoring. *Pseudomonas aeruginosa*, the causative pathogen of FN, accounts for approximately 15% of bloodstream infections and is associated with a high mortality rate [1]. Therefore, antibiotics with anti-*P. aeruginosa* activity should be administered appropriately during the initial treatment of FN.

Treatment approaches for FN vary among healthcare facilities, making adherence to established guidelines essential for improving patient outcomes [2]. Guidelines on FN have been published internationally since the 1990 s [3], and in Japan, guidelines on FN treatment were published by the Japanese Society of Medical Oncology in 2012, with subsequent editions released in 2017 and 2023 [4]. Japanese guidelines recommend monotherapy with beta-lactam antibiotics that have anti-*P. aeruginosa* activity, primarily cefepime, piperacillin/tazobactam (PIPC/TAZ), and meropenem, with no changes since 2012 [4]. Similarly, international guidelines also recommend monotherapy with beta-lactams, active against *P. aeruginosa* [5].

In the United States, a 2010 survey analyzing data from more than 600 hospitals reported a guideline adherence rate of 78.9% [2]. In addition, adherence to guideline-recommended antibiotic therapy has been associated with decreased mortality rates [2, 6]. Furthermore, surveys of factors related to guideline adherence have been conducted in other countries and have shown that physician expertise and healthcare facility conditions influence adherence [2, 7]. In Japan, the types of antibiotics used in the initial treatment of FN and the status of prophylactic administration of antifungal agents at a single institution have been examined [8]. Additionally, questionnaire-based surveys have assessed adherence to specific recommendations in Japanese FN guidelines [9, 10]. However, large-scale studies evaluating overall adherence to FN treatment guidelines and the factors influencing adherence in Japan remain limited.

This study aimed to determine the adherence status to FN treatment guidelines regarding antibiotics used in the initial treatment of FN in patients with cancer and identify factors associated with adherence using the health insurance claims database in Japan.

## Methods

### Data source

This study used the JMDC hospital-based administrative claims database, obtained from JMDC Inc., covering the period from April 2014 to September 2022. This database includes data from approximately 500 healthcare facilities, representing approximately 7.0% of all medical institutions in Japan. As it collects data regardless of patients’ insurance type, it also includes information on individuals aged > 65 years [11].

### Study period and patient selection

The study was conducted from April 2014 to September 2022. Hospitalized patients with cancer with a definitive diagnosis of FN were included. Patients without prescription data on the date of their initial FN diagnosis were excluded, as it was not possible to determine whether they actually had no prescription or had missing prescription data.

#### Definition

In this study, patients who underwent bacteriological culture and identification test for blood in the same month as their first definitive FN diagnosis were defined as those with a definitive diagnosis of FN. The database used in this study only provides diagnostic data at the monthly level, without detailed daily records. Therefore, the date of the first bacteriological culture and identification test for blood was defined as the date of the first definitive diagnosis of FN. Furthermore, patients who were prescribed antibiotics recommended in the guidelines on the date of the first definitive diagnosis of FN and continued to use them for 3 days were classified as the guideline adherence group. The antibiotics recommended in the Japanese FN guidelines are listed in Table 1.

**Table 1:** Antibiotic agents listed in the Japanese febrile neutropenia guidelines

Antibiotic group	Route	Ingredient name
Carbapenems	Parenteral	Biapenem
		Doripenem
		Imipenem/Cilastatin
		Meropenem
		Panipenem/Betamipron
Piperacillin with beta-lactamase inhibitor	Parenteral	Piperacillin/Tazobactam
Third-cephalosporins	Parenteral	Ceftazidime
Fourth-cephalosporins	Parenteral	Cefepime
		Cefozopran
		Cefpirome

In this study, FN, cancer, and comorbid conditions were defined using the Japanese disease codes and the International Classification of Diseases, 10 th edition (ICD-10). The specific codes used were as follows: FN (Japanese disease code: 8,842,350), cancer (ICD-10: C00-C97), hematologic cancer (ICD-10: C81-C96), pneumonia (ICD-10: J12-J18), and sepsis (ICD-10: A40, A41, P36, Japanese disease code: 8,841,319). Anti-methicillin-resistant Staphylococcus aureus (MRSA), antifungal, and antiviral agents were classified according to the Anatomical Therapeutic Chemical Classification System. The specific codes used were as follows: anti-MRSA agents (J01GB12, J01XA01, J01XA02, J01XX08, J01XX09, J01XX11), antifungal agents (J02), antiviral agents (J05). The receipt codes used in this study are listed in Supplemental Table 1.

### Statistical analysis

Univariate and multivariate logistic regression analyses were performed to determine the factors associated with guideline adherence. The following variables were included: age, sex, number of hospital beds, infection prevention and control premium [12], antimicrobial stewardship premium [12], Charlson Comorbidity Index, intensive care unit admission on the day of FN diagnosis, use of mechanical ventilation on the day of FN diagnosis, hematological malignancy, sepsis, and pneumonia. Changes in guideline adherence rates and antibiotics use rates over time were evaluated using the Cochran-Armitage test. After propensity score matching, the 28-day mortality rates in the guideline adherence and non-guideline adherence groups were compared using the chi-squared test. The propensity scores were calculated using logistic regression analysis and matched in a 1:1 ratio with a caliper of 0.2. The balance between the two groups was evaluated using the standardized mean difference (SMD), where an SMD < 0.10 indicated an appropriate balance of variables. Statistical significance was set at *P* < 0.05. Statistical analyses and data cleaning were performed using Stata version 18.0 (Stata Corp., College Station, TX, USA) and EZR (Saitama Medical Center, Jichi Medical University, Saitama, Japan) [13].

## Results

### Patient selection

**Fig. 1:**
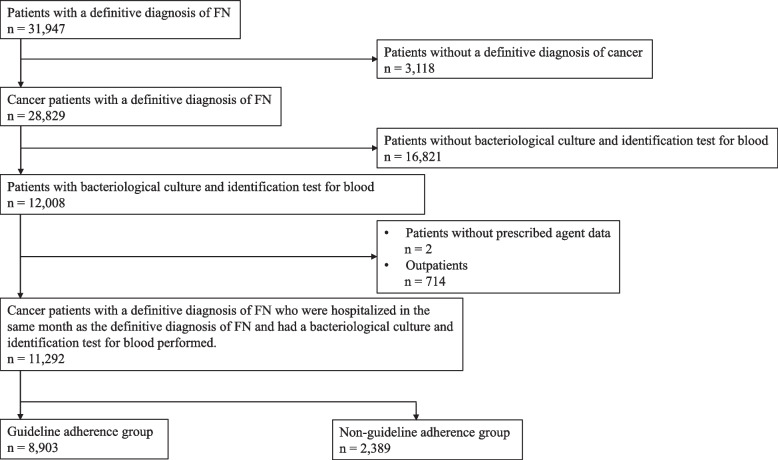
Patient selection

**Fig. 2:**
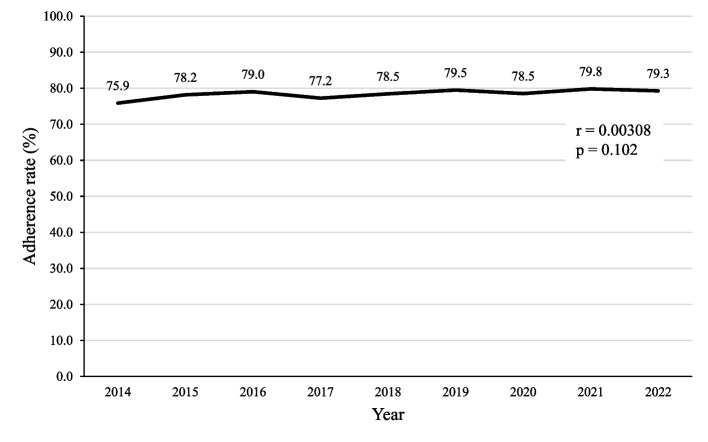
Trends in guideline adherence rates from 2014 to 2022

The patient selection process is summarized in a flowchart, as shown in Fig. 1. This database includes 31,947 patients with a definitive diagnosis of FN. Of these, 28,829 had cancer, and 12,008 underwent bacteriological culture and identification test for blood within the same month as their definitive FN diagnosis. After excluding patients with missing drug data and outpatients, 11,292 patients were included in the study. The guideline adherence group included 8,903 patients, resulting in an adherence rate of 78.8%. Trends in Guideline Adherence Rates from 2014 to 2022 are shown in Fig. 2. No significant changes in the guideline adherence rates over time were observed (*r* = 0.00308, *p* = 0.102).

### Evaluation of factors associated with guideline adherence

Patient characteristics for the guideline adherence and non-guideline adherence groups are shown in Table 2. The percentage of patients with hematologic malignancies was higher in the guideline adherence group (50.1%) than in the non-guideline adherence group (46.6%).

**Table 2:** Patient characteristics in the guideline adherence and non-guideline adherence group

	Guideline adherence group (*n* = 8,903)	Non-Guideline adherence group (*n* = 2,389)
Sex		
Man ^a^	5,347 (60.1%)	1,400 (58.6%)
Age ^b^	70 [61–76]	70 [61–77]
Number of beds ^a^		
≤ 199	537 (6.0%)	180 (7.5%)
200–499	4,076 (45.8%)	1,137 (47.6%)
500 ≤	4,290 (48.2%)	1,072 (44.9%)
Infection prevention and control premium ^a c^	4,536 (50.9%)	1,199 (50.2%)
Antimicrobial stewardship premium ^a^	3,007 (33.8%)	762 (31.9%)
CCI ^b^	4 [3–8]	4 [3–8]
ICU admission on the day of FN diagnosis ^a^	42 (0.5%)	20 (0.8%)
Use of mechanical ventilation on the day of FN diagnosis ^a^	39 (0.4%)	33 (1.4%)
Hematological malignancy ^a^	4,459 (50.1%)	1,113 (46.6%)
Pneumonia ^a^	1,668 (18.7%)	423 (17.7%)
Sepsis ^a^	1,638 (18.4%)	446 (18.7%)

*CCI* Charlson Comorbidity Index, *ICU* Intensive Care Unit, *FN* febrile neutropenia

^a^Data are expressed n (%)

^b^Data are expressed median [interquartile range]

^c^Additional reimbursement for infection prevention and Additional healthcare reimbursement for infection prevention and control

Table 3 shows the results of univariate and multivariate logistic regression analyses assessing factors associated with guideline adherence. Patients with hematologic malignancies (OR: 1.117, 95% CI: 1.007–1.239) were significantly associated with guideline adherence. Conversely, factors associated with guideline non-adherence included hospitalization in facilities with < 500 beds (500 ≤ vs. ≤ 199; OR: 0.775, 95% CI: 0.644–0.934, 500 ≤ vs. 200–499; OR: 0.898, 95% CI: 0.816–0.989) and the use of mechanical ventilation on the day of FN definitive diagnosis (OR: 0.317, 95% CI: 0.186–0.539).

**Table 3:** Factors associated with guideline adherence

	Univariate Analysis			Multivariate Analysis		
	OR	95% CI	*p* value	OR	95% CI	*p* value
Sex [woman vs. man]	1.062	0.969–1.164	0.197	1.081	0.985–1.187	0.100
Age (years)	0.998	0.994–1.001	0.150	0.998	0.995–1.001	0.246
Number of hospital beds						
[500 ≤ vs. ≤ 199]	0.745	0.622–0.894	0.002	0.775	0.644–0.934	0.007
[500 ≤ vs. 200–499]	0.896	0.816–0.984	0.022	0.898	0.816–0.989	0.029
Infection prevention and control premium ^a^	1.031	0.942–1.128	0.509	1.029	0.905–1.171	0.660
Antimicrobial stewardship premium	1.089	0.989–1.199	0.084	1.092	0.954–1.250	0.200
CCI	0.982	0.969–0.996	0.014	0.989	0.974–1.004	0.163
ICU admission on the day of FN diagnosis	0.561	0.329–0.958	0.034	0.976	0.520–1.831	0.939
Use of mechanical ventilation on the day of FN diagnosis	0.314	0.197–0.500	< 0.001	0.317	0.186–0.539	< 0.001
Hematological malignancy	1.150	1.051–1.259	0.002	1.117	1.007–1.239	0.037
Pneumonia	1.072	0.952–1.205	0.250	1.073	0.951–1.211	0.253
Sepsis	0.982	0.875–1.103	0.762	0.959	0.850–1.083	0.504

*OR* odds ratio, *95% CI* 95% confidence interval, *CCI* Charlson Comorbidity Index, *ICU* Intensive Care Unit, *FN* febrile neutropenia

^a^Additional reimbursement for infection prevention and healthcare reimbursement for infection prevention and control

### Prescribing status of antimicrobial agents on the day of definitive diagnosis of FN in the guideline group

The prescribing status of antimicrobial agents on the day of definitive diagnosis of FN in the guideline adherence group is shown in Table 4. In the guideline adherence group, 54.7% of patients received only the antibiotic agents recommended by the guidelines. Antifungal or antiviral agents were used on the date of FN definitive diagnosis in 33.1% of the cases. Additionally, 3.5% of patients received anti-MRSA agents on the day of FN definitive diagnosis.

**Table 4:** Antimicrobials administered on the date of the first definitive diagnosis of FN in the guideline adherence group

	*n* = 8,903
Only antimicrobials listed in the guidelines	4,866 (54.7%)
+ antifungal	1,450 (16.3%)
+ antiviral	396 (4.4%)
+ anti MRSA	100 (1.1%)
+ other	993 (11.2%)
+ antifungal, antiviral	890 (10.0%)
+ antifungal, anti MRSA	106 (1.2%)
+ antiviral, anti MRSA	11 (0.1%)
+ antifungal, antiviral, anti MRSA	91 (1.0%)

#### MRSA methicillin-resistant *Staphylococcus aureus*

Trends in the percentage of antibiotic agents listed in the FN guidelines on the date of definitive diagnosis for the guideline adherence group from 2014 to 2022 are shown in Table 5. The use of penicillin combined with beta-lactamase inhibitors significantly increased over time (*r* = 0.01621, *p* < 0.001). In contrast, carbapenem use significantly declined (*r* = −0.00813, *p* < 0.001).

**Table 5:** Trends in the use of antibiotics in the guideline adherence group from 2014 to 2022

	2014	2015	2016	2017	2018	2019	2020	2021	2022	Change	P _for trend_
Third-cephalosporins ^*1^	1.0	2.8	2.3	2.4	0.6	1.2	1.0	0.8	1.0	−0.00181	0.0011
Fourth-cephalosporins ^*2^	64.5	62.2	66.2	55.8	55.3	57.0	59.8	59.6	53.6	−0.00627	0.0137
Carbapenems ^*3^	26.8	23.7	17.1	21.2	22.5	19.5	18.2	18.0	18.5	−0.00813	< 0.001
Penicillin with beta-lactamase inhibitor ^*4^	7.7	11.2	14.4	20.5	21.6	22.3	21.0	21.7	27.0	0.01621	< 0.001

^*1^Ceftazidime

^*2^Cefepime, Cefozopran, Cefpirome

^*3^Biapenem, Doripenem, Imipenem/Cilastatin, Meropenem, Panipenem/Betamipron

^*4^Piperacillin/Tazobactam

### 28-day mortality

Patient characteristics for the guideline adherence and non-guideline adherence groups after propensity score matching are shown in Supplemental Table 2. After propensity score matching, 1,953 patients were matched in each group. Consequently, all factors had an SMD of < 0.001, and patient characteristics were adjusted. The 28-day mortality rate was significantly lower in the guideline adherence group than in the non-guideline adherence group (5.8% [113/1,953] vs. 8.6% [167/1,953]; *p* = 0.001) (Supplemental Figure 1).

## Discussion

This study is the first to determine guideline adherence rates and factors influencing adherence to FN guidelines in Japan using a health insurance claims database. The guideline adherence rate in Japan was 78.8% and remained stable over time, consistently above 75%. Given that FN treatment guidelines had already been published prior to the study period (2014–2022), it was suggested that the initial treatment of FN was well-established. In addition, adherence to the guidelines was validated to have an impact on 28-day mortality.

In the United States, the involvement of hematological malignancy and infectious disease specialists has been associated with increased adherence to FN antibiotic guidelines [7]. Consistent with previous findings, our study demonstrated a higher guideline adherence rate among patients with hematological malignancies. It is thought that patients with solid tumors are less likely to receive consultations from infectious disease specialists or antimicrobial stewardship programs, potentially contributing to lower adherence to the guidelines [7]. A similar trend was observed in this study. However, data on the involvement of infectious disease specialists were not collected from the Japanese claims data and, therefore, could not be analyzed as a factor in this study. As alternative indicators, we evaluated medical fees that could be claimed for facility functions such as the installation of infection control teams (since 2010) or antimicrobial stewardship teams (2018 to 2022). Consequently, although not significant, facilities with antimicrobial stewardship teams showed a tendency towards higher adherence to the guidelines (OR: 1.092, 95% CI: 0.954–1.250). In contrast, in Japan, medical fees are also assigned for each patient intervention, which is used to evaluate efficacy and safety [14]. Therefore, a new system will be required in the future to enable analysis with claims data not only of facility functions, but also of professional interventions for individual patients.

In this study, factors associated with guideline noncompliance were identified as < 500 beds and the use of mechanical ventilators on the date of FN definitive diagnosis. It has been reported that small and medium-sized hospitals do not have enough infectious disease specialists [15], and differences in knowledge and experience in infectious disease care may have an impact on guideline adherence rates. In patients with severe conditions, broad-spectrum antibiotics are often administered [16]. Therefore, in this study, indicators such as CCI, ICU admission on the day of FN diagnosis, use of mechanical ventilation on the day of FN diagnosis, hematologic malignancy, pneumonia, and sepsis were used to assess the severity of disease. However, the use of mechanical ventilation on the day of FN diagnosis was identified as a factor associated with non-guideline adherence. The reasons for this could not be clarified in this study. One possible explanation is that, due to the nature of database research, clinical information such as laboratory values and bacteriological culture results was not available [14]. Further investigations using data sources with more detailed clinical information, such as electronic medical records, are warranted.

Among patients in the guideline adherence group, 54.7% received only the recommended antibiotic agents listed in the guidelines on the date of definitive diagnosis of FN. However, it was found that antifungal, antiviral, and other agents were also administered on the date of FN definitive diagnosis in the other patients. Specifically, 33.1% of patients received antiviral or antifungal drugs on the date of FN definitive diagnosis. FN guidelines recommend prophylactic administration of antifungal and antiviral agents in patients at risk for severe neutropenia and in those receiving certain anticancer agents [4]. Given that 50.1% of patients in our study had hematologic malignancies, it is likely that antifungal or antiviral agents may have been administered prophylactically.

Anti-MRSA agents were used in 3.5% of patients on the day of definitive FN diagnosis, which is lower than previously reported rates [2]. According to FN guidelines, anti-MRSA agents should be considered when drug-resistant gram-positive cocci, such as MRSA, are suspected. Anti-MRSA agents were prescribed within 7 days in 9.0% of the patients who were not prescribed anti-MRSA agents on the date of FN definitive diagnosis (data not shown). Given the high mortality associated with MRSA infections [17, 18], early and appropriate initiation of anti-MRSA agents remains critical. However, due to the limitations of the database used in this study, patients’ detailed condition and culture results were not available. Therefore, a more detailed investigation should be conducted to evaluate the appropriateness of anti-MRSA agents use.

This study revealed significant temporal trends in antibiotic prescribing patterns and use. The use of penicillin with beta-lactamase inhibitor significantly increased, while carbapenem use significantly declined over time. Recently, the appropriate use of antimicrobial agents has been promoted worldwide, and the National Action Plan on Antimicrobial Resistance has been formulated in Japan. As a result, carbapenem resistance rates in *P. aeruginosa* and the use of intravenous antibiotic agents in Japan are declining [19]. The Guidelines for the Treatment of Infectious Diseases published by the Japanese Association for Infectious Diseases also recommend monotherapy with beta-lactams having anti-*P. aeruginosa* activity for empirical treatment of FN while cautioning against carbapenems overuse from an antimicrobial stewardship perspective [20]. The addition of FN to the indications for PIPC/TAZ in June 2015 may have contributed to this increase. Therefore, carbapenem-sparing therapy may also be administered to patients with FN.

The 28-day mortality rate was significantly lower in the guideline adherence group than in the non-guideline adherence group. Although direct comparisons are difficult owing to differences in study populations, previous studies have reported similar trends, with the guideline adherence group having a lower 28-day mortality rate than the non-guideline adherence group (guideline adherence group: 3.7%, non-guideline adherence group: 15.9%) [6]. Bloodstream infections are a major complication in patients with FN [21], and sepsis caused by *P. aeruginosa* carries a high mortality rate, underscoring the importance of initial *P. aeruginosa* coverage [22]. Previous studies indicate that adherence to guidelines may contribute to lower mortality rates [2, 6]. Similar to previous reports, the findings of this study showed a lower 28-day mortality rate in the guideline adherence group than in the non-guideline adherence group. Therefore, adherence to the guidelines for the initial antibiotic agent selection is very important for patients with FN.

This study has some limitations. First, the exact date of FN diagnosis was not obtained from the data used in this study. Therefore, in this study, the date of the first definitive diagnosis of FN was defined by combining the FN disease code and the bacteriological culture and identification test for blood within the same month. However, it is unclear whether the bacteriological culture and identification test for blood performed in the same month as the FN disease code were actually performed for FN. Furthermore, it is not possible to confirm that the necessary elements for the diagnosis of FN, fever, and neutropenia, were in temporal proximity to each other. Therefore, the date of the first definitive diagnosis of FN as defined in this study, may not match the timing of FN diagnosis in actual clinical practice. These points may have caused guideline adherence rates and antimicrobial prescribing trends to differ from actual results and need to be considered when interpreting the results. Second, the database used in this study was not collected from all medical institutions in Japan. Thus, it may not strictly reflect the actual situation in Japan as a whole. Despite these limitations, this study provides insights into the guideline adherence rate for FN treatment in Japan and the factors that influence adherence.

## Conclusion

The study revealed that the adherence rate to the FN guidelines in Japan is 78.8%, with notable changes in antibiotic use trends, including increased use of beta-lactamase inhibitors and decreased carbapenem use between 2014 and 2022. Given the potential impact of guideline revisions and ongoing antimicrobial stewardship efforts, continuous monitoring of adherence rates and prescribing patterns is necessary to optimize FN management and improve patient outcomes.

## Supplementary Information

Additional file 1. Comparison of 28-day mortality rates between the guideline adherence and non-guideline adherence groups.

Additional file 2. List of receipt codes used in this study. Patient characteristics and propensity score matching based on guideline adherence status.

## Data Availability

We purchased and used the data from JMDC. Therefore, these data are not publicly available. If other researchers wish to use the data, they need to purchase it, along with the authors.

## Abbreviations

FN: Febrile neutropenia
ICD-10: International Classification of Diseases, 10th edition
MRSA: Methicillin-resistant Staphylococcus aureus
PIPC/TAZ: Piperacillin/tazobactam
SMD: Standardized mean difference
