# Supplementary material for: Adherence to guidelines for antibiotics used in the initial treatment of febrile neutropenia in patients with cancer: a study using health insurance claims database in Japan
Source: J Pharm Health Care Sci. 2025 Jun 6;11:47. doi: 10.1186/s40780-025-00455-0 (PMC12144810; doi:10.1186/s40780-025-00455-0)
Supplement: Supplementary file 1 — Additional file 1. Comparison of 28-day mortality rates between the guideline adherence and non-guideline adherence groups. [file 40780_2025_455_MOESM1_ESM.pptx]

## Slide 1
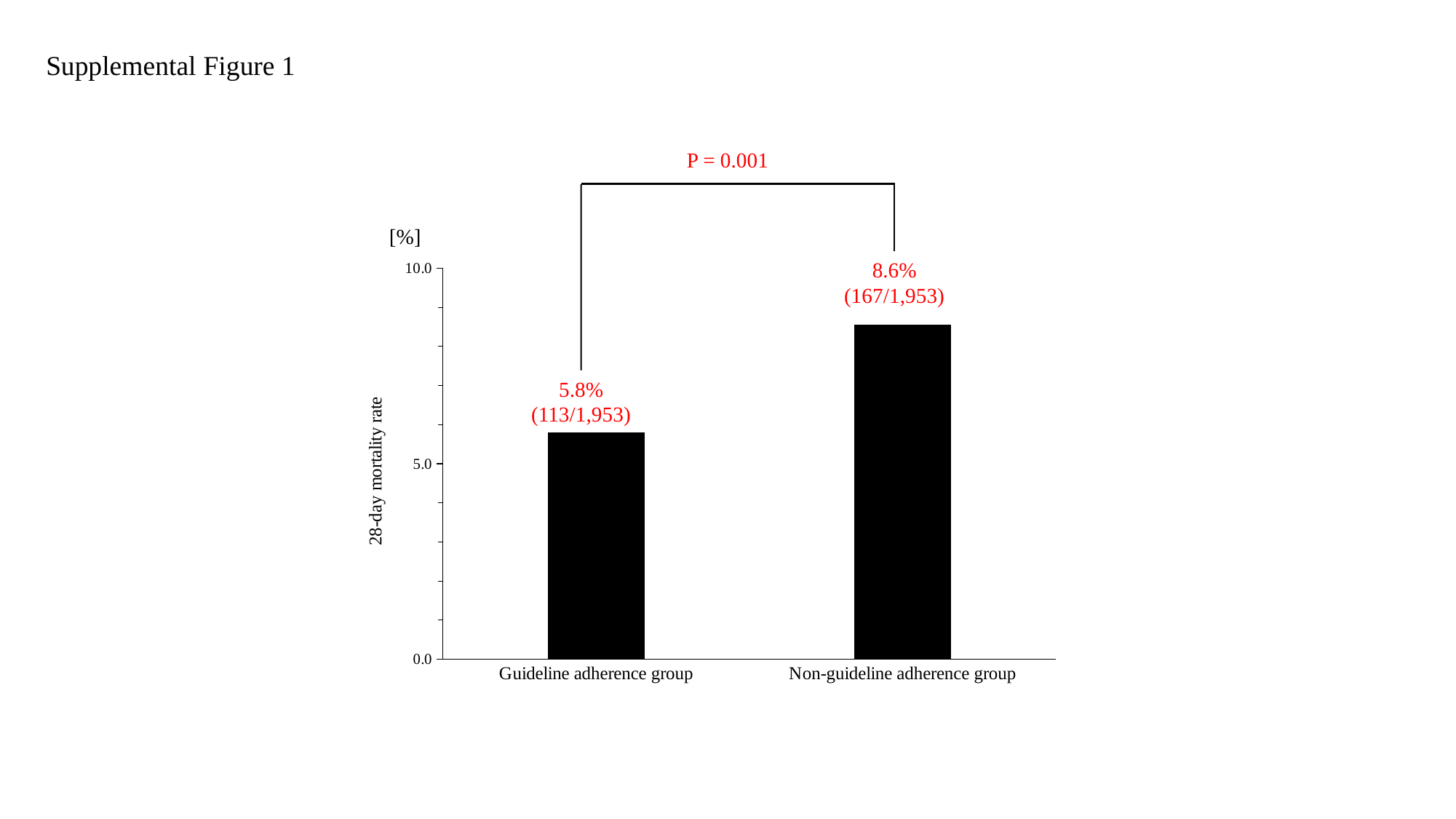

Supplemental Figure 1
P = 0.001
[%]
### Chart
| Category | 28日死亡率 |
|---|---|
| Guideline adherence group | 5.785970302099335 |
| Non-guideline adherence group | 8.55094726062468 |8.6%
(167/1,953)
5.8%
(113/1,953)
