# Supplementary material for: Adherence to guidelines for antibiotics used in the initial treatment of febrile neutropenia in patients with cancer: a study using health insurance claims database in Japan
Source: J Pharm Health Care Sci. 2025 Jun 6;11:47. doi: 10.1186/s40780-025-00455-0 (PMC12144810; doi:10.1186/s40780-025-00455-0)
Supplement: Supplementary file 2 — Additional file 2. List of receipt codes used in this study. Patient characteristics and propensity score matching based on guideline adherence status. [file 40780_2025_455_MOESM2_ESM.docx]

|  | Receipt code | | | | |
| --- | --- | --- | --- | --- | --- |
| Bacteriological culture and  identification test for blood | 160058610 |  |  |  |  |
| Additional reimbursement  for infection prevention 1, 2 | 190147210 | 190147310 |  |  |  |
| Additional healthcare reimbursement  for infection prevention and control 1, 2, 3 | 190147210 | 190147310 | 190243810 |  |  |
| Antimicrobial stewardship premium | 190206870 |  |  |  |  |
| ICU admission | 190024710 | 190066710 | 190066810 | 190116310 | 190116410 |
|  | 190137610 | 190139810 | 190139910 | 190140270 | 190140370 |
|  | 190140410 | 190148410 | 190148510 | 190174410 | 190174510 |
|  | 190174610 | 190174710 | 190223050 | 190223150 | 190223250 |
|  | 190223350 | 190223450 | 190223550 | 190227150 | 190227250 |
|  | 190227350 | 190227450 | 190227550 | 190227650 | 190227750 |
|  | 190227850 | 190228550 | 190228650 | 190228750 | 190230610 |
|  | 190235210 | 190235310 | 190246510 | 190246610 | 190246810 |
|  | 190246910 | 190247110 | 190247210 |  |  |
| Mechanical ventilation | 140009310 | 140009550 | 140009650 | 140009750 | 140009850 |
|  | 140009950 | 140010050 | 140010150 | 140023510 | 140023650 |
|  | 140023750 | 140023850 | 140023950 | 140024050 | 140024150 |
|  | 140024250 | 140024350 | 140039550 | 140039650 | 140039850 |
|  | 140039950 | 140051750 | 140055650 | 140056170 | 140056270 |
|  | 140063310 | 140063410 | 140063810 | 140064050 | 140064150 |
|  | 140064250 | 140064350 | 140064550 | 140064750 |  |

Supplemental Table 1. List of receipt codes used in this study.

Supplemental Table 2. Patient characteristics and propensity score matching based on guideline adherence status.

|  | Before propensity score matching | | | After propensity score matching | | |
| --- | --- | --- | --- | --- | --- | --- |
|  | Guideline adherence  group  (n = 8,903) | Non-Guideline adherence  group  (n = 2,389) | SMD | Guideline adherence  group  (n = 1,953) | Non-Guideline adherence  group  (n = 1,953) | SMD |
| Sex |  |  |  |  |  |  |
| Man ^a^ | 5,347 (60.1%) | 1,400 (58.6%) | 0.030 | 1,155 (59.1%) | 1,155 (59.1%) | <0.001 |
| Age ^b^ | 70 [61-76] | 70 [61-77] | 0.033 | 70 [63-76] | 70 [63-77] | <0.001 |
| Number of beds ^a^ |  |  | 0.080 |  |  | <0.001 |
| ≤ 199 | 537 (6.0%) | 180 (7.5%) |  | 105 (5.4%) | 105 (5.4%) |  |
| 200-499 | 4,076 (45.8%) | 1,137 (47.6%) |  | 953 (48.8%) | 953 (48.8%) |  |
| 500 ≤ | 4,290 (48.2%) | 1,072 (44.9%) |  | 895 (45.8%) | 895 (45.8%) |  |
| CCI ^b^ | 4 [3-8] | 4 [3-8] | 0.057 | 4 [3-8] | 4 [3-8] | <0.001 |
| ICU admission on the day of  FN diagnosis ^a^ | 42 (0.5%) | 20 (0.8%) | 0.045 | 3 (0.2%) | 3 (0.2%) | <0.001 |
| Use of mechanical ventilation  on the day of FN diagnosis ^a^ | 39 (0.4%) | 33 (1.4%) | 0.099 | 2 (0.1%) | 2 (0.1%) | <0.001 |
| Hematological malignancy ^a^ | 4,459 (50.1%) | 1,113 (46.6%) | 0.070 | 921 (47.2%) | 921 (47.2%) | <0.001 |
| Pneumonia ^a^ | 1,668 (18.7%) | 423 (17.7%) | 0.027 | 265 (13.6%) | 265 (13.6%) | <0.001 |
| Sepsis ^a^ | 1,638 (18.4%) | 446 (18.7%) | 0.007 | 249 (12.7%) | 249 (12.7%) | <0.001 |

SMD standardized mean difference, CCI Charlson Comorbidity Index, ICU Intensive Care Unit, FN febrile neutropenia

^a^ Data are expressed n (%)

^b^ Data are expressed median [interquartile range]
